# Distinct neurocognitive fingerprints reflect differential associations with risky and impulsive behavior in a neurotypical sample

**DOI:** 10.1038/s41598-023-38991-0

**Published:** 2023-07-21

**Authors:** Sonia G. Ruiz, Inti A. Brazil, Arielle Baskin-Sommers

**Affiliations:** 1 Department of Psychology, Yale University, New Haven, CT USA; 2 Donders Institute for Brain, Cognition and Behaviour, Radboud University, Nijmegen, The Netherlands; 3 Forensic Psychiatric Centre Pompestichting, Nijmegen, The Netherlands

**Keywords:** Psychology, Human behaviour

## Abstract

Engagement in risky and impulsive behavior has long been associated with deficits in neurocognition. However, we have a limited understanding of how multiple subfunctions of neurocognition co-occur within individuals and which combinations of neurocognitive subfunctions are most relevant for risky and impulsive behavior. Using the neurotypical Nathan Kline Institute Rockland Sample (N = 673), we applied a Bayesian latent feature learning model—the Indian Buffet Process—to identify nuanced, individual-specific profiles of multiple neurocognitive subfunctions and examine their relationship to risky and impulsive behavior. All features were within a relatively normative range of neurocognition; however, there was subtle variability related to risky and impulsive behaviors. The relatively overall poorer neurocognition feature correlated with greater affective impulsivity and substance use patterns/problems. The poorer episodic memory and emotion feature correlated with greater trait externalizing and sensation-seeking. The poorer attention feature correlated with increased trait externalizing and negative urgency but decreased positive urgency and substance use. Finally, the average or mixed features negatively correlated with various risky and impulsive behaviors. Estimating nuanced patterns of co-occurring neurocognitive functions can inform our understanding of a continuum of risky and impulsive behaviors.

## Introduction

Risky and impulsive behaviors, including substance use, physical aggression, and other health-risk behaviors, are common. In 2019, 50.8% of Americans reported using alcohol^1^. Among these individuals, 47.1% binge drank and 11.5% engaged in heavy alcohol use within the past month. In that same year, a violent crime was committed every 26.3 s in the United States^2^. Further, rates of substance use and aggression rose sharply during the COVID-19 pandemic. Thirteen percent of Americans reported starting or increasing substance use to cope with pandemic-related stress^3^. Homicide rates rose by 4% after the onset of the pandemic and remained elevated through 2021^4^. Other health-risk behaviors also were a prominent feature of the pandemic. In January 2022, over 40% of Americans reported non-compliance with COVID preventative measures within the past week (e.g., wearing masks, hand-washing)^5^. Altogether, these behaviors exacted profound costs on society by increasing the likelihood of premature death, long-term disability, and poor mental health outcomes^6–9^. To understand who tends most toward these risky and impulsive behaviors, much work has focused on neurocognitive functions that support the initiation, planning, representation, and achievement of flexible, goal-directed behaviors^10^.

Extant work suggests that deficits in various neurocognitive functions are linked to engagement in risky and impulsive behavior^11–16^. For example, deficits in working memory (the maintenance and manipulation of information) are associated with sustained substance use. This relationship suggests that difficulties retaining knowledge of use-related consequences (i.e., maintaining information) and applying this knowledge to new situations (i.e., manipulating information) may inform poorer decision-making about substance use^17,18^. Additionally, difficulties sustaining attention to goal-relevant stimuli, particularly at high levels of distraction, are related to trait impulsivity^19,20^. This relationship has been interpreted to indicate that difficulties processing goal-relevant cues may contribute to rash decision-making across contexts. Finally, deficits in response inhibition and cognitive control relate to a latent construct of externalizing (which encompasses trait impulsivity and risky behaviors such as substance misuse), suggesting that a general difficulty inhibiting inappropriate responses while selecting goal-relevant actions underlies the tendency for individuals high in externalizing to display risky and impulsive behavior^21,22^. Overall, there is substantial evidence that supports the general relationship between neurocognitive deficits and risky and impulsive behaviors^13,16^.

However, much of this evidence is derived from work that measures neurocognition as isolated or separable subfunctions^13,16,22–25^, despite evidence that behavior is supported by interactions among neurocognitive subfunctions^26–28^. For example, successful inhibition of responses to irrelevant stimuli (i.e., response inhibition) may rely on sustained attention to relevant information (i.e., attention) as well as maintenance of this information (i.e., working memory)^13^. As a result, studies that look at performance on a single task^17–22^ are unable to account for the potential influence of other subfunctions. Similarly, studies that use a battery of tasks^26–33^ tend to treat each task as an independent measure of neurocognition, rather than capturing the interplay among neurocognitive subfunctions that are known to occur within individuals. Thus, it remains to be demonstrated how different subfunctions of neurocognition co-occur within individuals and how these may relate to different forms of risky and impulsive behavior. For instance, certain subfunctions, either individually or in concert, may be related to specific forms of risky and impulsive behavior or might exert stronger influences on behavior in some individuals and not in others. Therefore, comprehensively estimating neurocognitive subfunctions, examining their relative co-occurrence within individuals, and then specifying their relations to risky and impulsive behaviors might allow for a more nuanced representation of who engages in these behaviors and which subfunctions contribute to that behavior.

In the present study, we applied a novel Bayesian latent feature model, Indian Buffet Process (IBP), to identify individual-specific profiles of co-occurring neurocognitive subfunctions to more precisely characterize their relationship to risky and impulsive behavior. Briefly, IBP is a Bayesian non-parametric dimension reduction method that derives latent features representing patterns of data for each individual^34^. IBP has been used in cognitive science research to build Artificial Intelligence models of images that allow for recognition or classification, or to represent generalizable sensory-derived information^34–36^. A major advantage of IBP is that it derives latent features based on patterns seen across the data, including across tasks^34,35^. Consequently, neurocognition would not be described as separable subfunctions of working memory, attention, or inhibition. Instead, neurocognition would be described as how an individual distributes the deployment of resources across individual neurocognitive subfunctions to extract the relevant pieces of information across experimental contexts (i.e., contexts that involve errors to detect, distracting cues to filter, or important consequences to remember)^37^.

Data were from the Nathan Kline Institute Rockland Sample. We used a sample that is characterized as neurotypical to test whether IBP could capture the patterns of neurocognition without clearly deficient, or extreme, performance. Because risky and impulsive behavior occurs along a continuum, and because it is unlikely that categorically “deficient” neurocognition contributes to this behavior, examining both risky behavior and neurocognition within a relatively normative range is a strong test of the application of IBP for extracting potentially meaningful variation in cognition and behavior as it relates to risky and impulsive behaviors.

Our analyses reflect a combination of a theory- and data-driven approach to identify relevant relationships between neurocognition and risky/impulsive behaviors. First, we applied IBP to 27 measures of neurocognitive function from the Nathan Kline Institute Rockland Sample. The selection of neurocognitive measures was based on previous theory and empirical work relevant to risky and impulsive behavior^11,12,14–22^. Second, we used dependent correlations to specify the relationship between the resulting features and different measures of risky/impulsive behavior. Risky and impulsive behavior can be measured in correlated, but distinct, ways from general traits (impulsivity; externalizing) to specific behaviors (substance use). We examined multiple measures of risky and impulsive behaviors to explore whether some latent features related to risky and impulsive behaviors in general or specific forms.

True to the flexible nature of IBP and following from a data-driven approach, we expected that several latent features would emerge from IBP, each representing different patterns of function across the 27 measures. Consistent with prior research, we hypothesized that latent features representing poorer neurocognitive function would correlate with more risky and impulsive behaviors relative to features representing better neurocognitive functioning. Given that this study presents a novel application of IBP, we did not have specific hypotheses about the relationships between additional features and risky/impulsive behaviors.

## Methods

### Participants

Participants were adults from the nationally representative Nathan Kline Institute Rockland Sample (NKI-RS), a community-ascertained sample recruited from Rockland County, New York; for details on recruitment method see Nooner et al.^38^. All procedures in the present study were approved by the Institutional Review Board at the Nathan Kline Institute and were performed in accordance with relevant guidelines. As part of the Nathan Kline Institute’s study procedure for this dataset, informed consent was obtained from all participants and research was performed in accordance with the Declaration of Helsinki. Among 1488 subjects with available phenotypic data as of December 2021, participants between ages 18 and 55 years were included. Participants (1) missing data on age or (2) under 18 and over 55 were excluded due to low rates of substance use and to constrain analyses to externalizing during young through middle adulthood. No other exclusion criteria were applied to the sample. The study sample included 673 participants (Age: 37.03 ± 11.95; 64% Female, 36% Male; 67% White, 21% Black, 6% Asian, 4% Other, 1% American Indian/Native Alaskan, 1% Native Hawaiian/Pacific Islander; 14% Hispanic; < 1% completed junior high school, 1% partially completed high school, 11% graduated high school, 36% partially completed college, 29% graduated college or university, 20% completed a graduate degree, and 2% were missing data on highest education completed). The Bayesian approach used in our analysis does not require a predetermined minimum amount of data to be used or distribution of data^39^. Rather, results are accompanied by estimates of uncertainty, which are conditioned on the available data^41^. However, this sample is larger than other studies using NKI-RS data to investigate risky and impulsive behavior, including substance use^40^.

### Measures

#### Neurocognitive functioning

To capture a broad array of neurocognitive functioning across subfunctions and modalities, we used behavioral data from 27 neurocognitive tasks included in the Nathan Kline Institute battery. As our aim was not to validate separate neurocognitive batteries, we selected measures that reflected overlapping subfunctions (e.g., error detection in planning, inhibition, and cognitive control) while limiting redundancy (e.g., selecting one measure of working memory in which sequences of characters are repeated). Neurocognitive tasks were administered via paper and pencil or computer^38^. Correlations across sample demographics and neurocognitive measures are provided in Fig. S1a.

##### Delis–Kaplan Executive Function System

The Delis–Kaplan Executive Function System (D-KEFS) is a nationally standardized and age-normed battery of nine standalone tests that evaluate key subfunctions of executive functioning (e.g., planning, inhibition, cognitive flexibility, working memory)^42^. The nine D-KEFS tests measure verbal and non-verbal executive functions^43^. A variety of age-normed primary scores, contrast scores, and cumulative percentile ranks are provided for each test, with higher values reflecting better performance. D-KEFS tests demonstrate moderate test–retest reliability, consistent with other measures of executive functioning^42,44^. We considered tests containing over 50% complete data and selected scores corresponding to a variety of neurocognitive subfunctions (e.g., cognitive flexibility in the visual and verbal modalities; Table 1).

**Table 1:** Summary of neurocognitive tests used in the present study.

Test	Neurocognitive subfunction	Score used
Delis–Kaplan Executive Function System		
Twenty Questions	Abstract categorization, visual attention, and perception	Total Questions Asked
Design Fluency	Cognitive flexibility and inhibition in the visual and motor modalities	Total Correct
Proverb	Verbal abstraction	Total Achievement
Tower	Spatial planning and rule learning	Total Achievement
	Spatial planning and error detection as relevant to planning	Rule Violations Per Item Ratio
Trail-Making	Attention	Number Sequencing
	Cognitive flexibility in the visual modalitiy	Number-Letter Switching
Verbal Fluency	Cognitive flexibility in the verbal modality	Category Switching
Color-Word Interference Test	Inhibition adjusted for basic naming skills	Inhibition-Color Naming
	Cognitive flexibility adjusted for basic naming skills	Inhibition/Switching-Color Naming
	Error detection related to inhibition	Inhibition uncorrected error
	Error detection related to cognitive control	Inhibition/Switching uncorrected error
Attention Network Test		
Alerting, orienting, and executive attention	Three components of attention and inhibition	Time-based efficiency scores for alerting, orienting and executive attention
Penn Computerized Neurocognitive Battery		
Mouse Practice Test	Sensorimotor processing speed	Efficiency score
Continuous Performance Test	Sustained attention	Efficiency score
Conditional Exclusion Test	Abstraction and cognitive flexibility	Efficiency score
Emotion Differentiation	Social cognition	Efficiency score
Emotion Identification	Social cognition	Efficiency score
Word Memory Test	Episodic memory in the verbal modality	Efficiency score
Visual Object Learning Test	Episodic memory in the spatial modality	Efficiency score
Verbal Reasoning Test	Language reasoning	Efficiency score
Finger Tapping Test	Sensorimotor processing speed	Sum score for mean dominant hand and non-dominant hand taps
Digit Span		
Digit Forwards	Attention and working memory	Raw score for longest length forward
Digit Backwards	Working memory	Raw score for longest length backwards
Rey Auditory Verbal Learning Test		
Delayed Recall	Learning and memory	Total correct scores

All neurocognitive task scores were standardized (z-score) prior to inclusion in the Indian Buffet Process dimension reduction procedure.

##### Attention Network Test

The Attention Network Test (ANT) is a computerized task developed to assess three components of attention: alerting (the ability to achieve and maintain an attentive state), orienting (the ability to select relevant information from sensory inputs), and executive attention (the ability to resolve conflict among responses, i.e., distractor stimuli)^45^. To tap these components of attention, the ANT makes use of alerting cues, spatial cues, and flanker arrows adapted from the widely-used Posner cueing task and Eriksen flanker task^46^. The ANT demonstrates acceptable reliability^45^. Scores from the ANT were reverse scored so that higher scores reflected better performance (Table 1).

##### Penn Computerized Neurocognitive Battery

The Penn Computerized Neurocognitive Battery (CNB) is a battery of 14 tests that provides estimates of efficiency (accuracy proportional to speed) across nine subfunctions of neurocognition: abstraction, attention, cognitive flexibility, emotion identification, episodic memory, language, sensorimotor speed, spatial processing, and working memory^47^. Derived from functional neuroimaging tasks, CNB measures of functioning across these subfunctions have been linked to specific brain systems. The CNB demonstrates moderate to high internal consistency (Cronbach’s αs = 0.55 to 0.98), validity, and moderate to high reliability^48,49^. Of the eleven available tests, we selected eight containing over 50% complete data and assessing subfunctions besides those assessed in D-KEFS (Table 1). In line with previous research, scores on the CNB Finger Tapping task that were fewer than 10 taps or more than 151 taps (*n* = 22) were replaced as missing, as they likely reflected technical issues (e.g., scores capture the average number of taps across five 10-s periods combined from both dominant and non-dominant hands)^50^.

##### Digit Span

Digit Span is a subtest of the nationally standardized and age-normed Weschler Adult Intelligence Scale-Revised (WAIS-R)^51^. Digit Span is used to assess attention and working memory, measured through the ability to recall strings of verbally presented numbers in the same order (Digit Forwards) and in backwards order (Digit Backwards; Table 1). Digit Forwards and Digit Backwards scores indicate acceptable split-half reliability (Cronbach’s α = 0.82 and 0.83, respectively)^52^.

##### Rey Auditory Verbal Learning Test

The Rey Auditory Verbal Learning Test (RAVLT) is a neuropsychological test that assesses learning and memory^53^. Immediate and delayed memory are captured through immediate and delayed (after 20 min) recall of all items remembered from 15-item word lists verbally presented over 5 trials (Table 1). RAVLT scores demonstrate acceptable internal consistency (Cronbach’s α = 0.80) and test–retest reliability (summed trials r = 0.68)^54^.

#### Risky/impulsive behavior and substance use outcomes

Risky and impulsive behavior, as well as substance use patterns, were estimated using personality, behavior, and clinical screening tools^38^. See Fig. S2 for outcome measure distributions.

##### UPPS-P Impulsive Behavior Scale

The UPPS-P is a 59-item self-report questionnaire assessing the tendency to engage in impulsive behavior under five conditions: Positive Urgency, Negative Urgency, Lack of Premeditation, Lack of Perseverance, and Sensation Seeking^20^. For each item, participants consider their behavior in the past 6 months and indicate whether they Agree Strongly (1), Agree Some (2), Disagree Some (3), or Disagree Strongly (4). Scores for each subscale are summed, such that higher scores indicate greater impulsivity.

##### Achenbach System of Empirically Based Assessment Adult Self Report

The Adult Self Report (ASR) is a 126-item self-report questionnaire that assesses behavior problems in adults (ages 18–59)^55^. The ASR has been standardized and provides *t*-scores for a variety of subscales nationally normed by age and sex. Here, the externalizing *t*-score was used to estimate aggressive, rule-breaking, and intrusive behavior, with higher scores indicating more externalizing behavior.

##### Comprehensive Addiction Severity Index

The Comprehensive Addiction Severity Index—Alcohol and Other Drugs (CASI-AOD) module was administered to participants ages 13–85 years old^56^. The CASI is an interview-based screening tool with modules spanning areas of functioning such as general health, mental health, and use of alcohol and other drugs. The CASI-AOD assesses multiple aspects of substance use (i.e., age of first use, use frequency in the past month) for several categories of drugs. In the present study, typical and peak use patterns in the past year as well as use frequency in the past month for alcohol, cannabis, and tobacco were used to characterize substance use patterns, with higher scores indicating greater use.

##### National Institute on Drug Abuse Quick Screen

The National Institute on Drug Abuse (NIDA) Quick Screen V1.0. is a brief 8-item self-report questionnaire assessing use and negative consequences stemming from use of a variety of substances^57^. Items for each substance are summed into Substance Involvement scores reflecting the level of risk for substance use disorder associated with each substance. Scores ranging from 0 to 3 reflect low risk, scores 4–26 reflect moderate risk, and scores above 27 reflect high risk. Here, Substance Involvement scores for alcohol, cannabis, and tobacco were used to characterize substance use problems.

### Data analysis

#### Indian Buffet Process

We used the Indian Buffet Process (IBP) to reduce the 27 neurocognitive variables of interest into combinations of latent features via the open-source Python Indian Buffet Process package (PyIBP)^58^. IBP has advantages over other analytic techniques used in research on risky and impulsive behaviors for two reasons. First, IBP addresses the “correct number” problem. The analytic approaches typically used to characterize neurocognition in relation to risky and impulsive behavior require specifying a “correct number” of parameters beforehand (e.g., single predictors in a typical regression or factors/classes of neurocognitive functions) so that the model can converge or can be selected^34^. Bayesian non-parametric models offer one solution to the problem of “correct numbers” by drawing a subset of parameters (factors, classes, functions) from an infinite-dimensional parameter space, which allows for flexibility in the number of parameters of interest. IBP, for example, determines the number of latent features based on the conditional distribution of latent (unobservable) features given the observed data, which allows the number of features to grow or shrink depending on the observed data. Here, conditioning the number of features on the observed data provides a flexible model of neurocognitive function that captures subtle variability across tasks in features that may have otherwise been collapsed together. Second, as previously mentioned, IBP extracts latent features based on patterns seen across the data, including across tasks that span various modalities and subfunctions^34,35,37^. Consequently, neurocognition is not estimated as separable subfunctions, but rather as co-occuring subfunctions within individuals. Altogether, IBP presents an opportunity to represent patterns of neurocognitive function in latent features, while preserving variability for each individual.

IBP can be described with the following culinary metaphor. Imagine that *N* customers (observations) sequentially enter a buffet with an infinite number of dishes (features). The first customer samples Poisson(*α*) number of dishes during their first sweep of the buffet. Each following *i*th customer samples the previously sampled dishes with a probability proportional to its existing popularity ($\left(\frac{{m}_{k}}{i}\right)$, where *m*_*k*_ is the number of previous customers who already sampled dish *k* and *i* is the number of customers so far), and samples new (i.e., previously unsampled) dishes with probability Poisson $\left(\frac{\alpha }{i}\right)$. In the resulting solution, *K* independent latent features (i.e., dishes) are responsible for generating (i.e., are sampled by) *N* observations (i.e., customers’ observed data), with flexibility. That is, sampling one latent feature does not inform whether an observation samples another latent feature, and the number of latent features will grow or shrink to reflect the data. The concentration parameter *α* governs the number of features sampled and the likelihood that observations will share the same features, such that larger values of *α* result in more features being sampled and shared. We used a variant of the IBP that models uncertainty regarding the number of features sampled, in which continuous values (rather than binary) represent the *extent* to which each feature is sampled.

The PyIBP package utilizes accelerated Gibbs sampling for a linear Gaussian model, which assumes that the observed data is drawn from a normal distribution. Because the Gibbs sampler accounts for missing data (i.e., removing data points from the posterior mean to determine their influence on the sampled latent features), we did not impute or exclude missing data. Completeness for each of the 27 neurocognitive variables, impulsive behavior, and substance use outcomes is documented in Table S1. The Gibbs sampling procedure also helps the model converge with fewer iterations^36,41^.

In the present study, the N observations are the 673 participants. Scores from the 27 neurocognitive measures spanning subfunctions and modalities of neurocognitive function were z-scored prior to the initiation of IBP. The resulting solution offers K latent features that represent patterns of performance seen across these data. Specificity is retained for each participant through the unique combination of sampled latent features (influenced by the concentration parameter *α*), and the extent to which each latent feature is sampled (i.e., their modeled uncertainty). Therefore, IBP provides a novel approach of identifying overall patterns (represented by features) across measures and extracting variability in those patterns to identify how individuals may engage neurocognitive functions. The concentration parameter *α* was initialized at five. For each participant, we extracted the continuous feature values corresponding to their unique combination of sampled latent features (*K*) present in the final iteration, where higher values indicate greater expression of a feature. With IBP, individuals may sample multiple features, allowing individuals to vary in their combinations of features. ANOVAs and Chi-Square tests of independence assessed whether the feature membership differed in terms of age, sex, or race/ethnicity.

#### Dependent correlations

Continuous feature value correlation analyses were conducted to assess how each latent feature—representing patterns of neurocognitive function—related to risky/impulsive behavior and substance use outcomes. Pearson correlations were performed in R version 4.1.1 to probe the relationship between feature values and outcomes^59^. Differences in the magnitude of correlations between feature values and outcomes were assessed using tests of dependent correlations performed via the R package *cocor*^60–62^. Tests of dependent correlations accounted for the potential overlap captured by the continuous feature value (i.e., probability that someone sampled a given latent feature and individuals could sample multiple latent features). Bonferroni correction accounted for the number of measures used to assess risky/impulsive behavior (*n* = 2) and substance use (*n* = 4).

## Results

### Latent features represent neurocognitive patterns

Traceplots demonstrated model convergence at 10 iterations (Fig. S3). *K*, the number of latent features, oscillated between 22 and 23 and stabilized at 22; the concentration parameter *α* remained between 1.7 and 2.3 and stabilized at 1.7; *σ*_*X*_ (the variance of the observed data) stabilized at 0.9; *σ*_*A*_ (the variance of the posterior mean weights) stabilized at 0.4. Feature sampling counts are detailed in Table S2.

**Figure 1:**
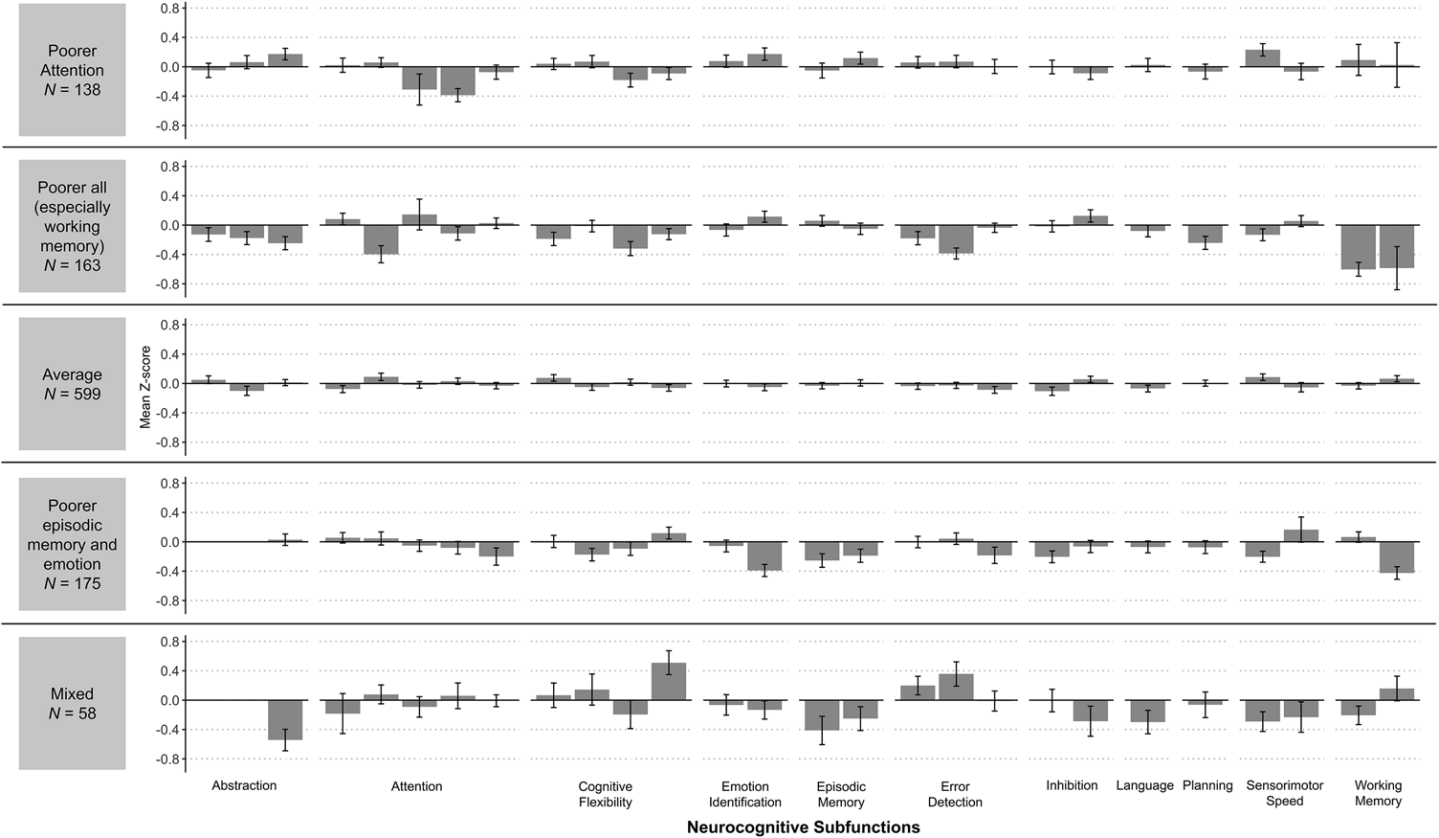
Neurocognitive patterns represented by latent features. Mean z-score and counts for each of the 27 neurocognitive measures across individuals who sampled each latent feature. Individual measures corresponding to each subfunction, ordered left to right, are as follows: Abstraction (D-KEFS Twenty Questions: Total Questions Asked; D-KEFS Proverb: Total Achievement; CNB Conditional Exclusion Test: Efficiency), attention (ANT Alert Efficiency; ANT Orienting Efficiency; Digit Span Forward Longest Length; D-KEFS Trail-Making: Number Sequencing; CNB Continuous Performance Test: Efficiency), cognitive flexibility (D-KEFS Color-Word Interference: Inhibition/Switching-Color Naming; D-KEFS Design Fluency: Total Correct; D-KEFS Trail-Making Test: Number-Letter Switching; D-KEFS Verbal Fluency: Category Switching), emotion (CNB Emotion Differentiation: Efficiency; CNB Emotion Recognition: Efficiency), episodic memory (CNB Word Memory: Efficiency; CNB Visual Object Learning Test: Efficiency), error detection (D-KEFS Color-Word Interference: Inhibition Total Errors Uncorrected; D-KEFS Color-Word Interference: Inhibition/Switching Total Uncorrected Errors; D-KEFS Tower: Rule Violations Per Item), inhibition (ANT Executive Attention Efficiency; D-KEFS Color-Word Interference: Inhibition-Color Naming), language (CNB Verbal Reasoning: Efficiency), planning (D-KEFS Tower: Total Achievement), sensorimotor speed (CNB Mouse Practice: Efficiency; CNB Finger Tapping: Total Taps), working memory (Digit Span Backwards Longest Length; RAVLT Delay Total Correct). *D-KEFS* Delis-Kaplan Executive Function System, *ANT* Attention Network Test, *CNB* Penn Computerized Neurocognitive Battery, *RAVLT* Rey Auditory Verbal Learning Test.

Though the resulting latent features are individually informative, it is difficult to extract generalizable group conclusions from features with few individuals (i.e., one or two). In line with previous work^63,64^, we only examined features with counts of at least 5% of the total sample size to appropriately represent how participants sampled features. Therefore, we selected features with at least 33 people to examine in the analyses. Five features met this criterion: (i) One feature was characterized by relatively poorer attention; (ii) one feature was characterized by relatively poorer functioning across neurocognitive subfunctions, particularly working memory; (iii) one feature captured average functioning across neurocognitive subfunctions; (iv) one feature was characterized by poorer episodic memory and emotion identification; and (v) one feature captured a variety of functioning, including poorer abstraction and episodic memory but better cognitive flexibility and error detection (Fig. 1; z-score means and standard errors are detailed in Table S3). Feature membership did not differ based on age, sex, or race/ethnicity, all *p*s > 0.1. See Table S4 for multi-feature sampling counts and descriptions. Finally, see Supplemental Material for follow-up analyses indicating that the neurocognitive patterns presented here held when IBP was applied to half the sample.

### Relationships between neurocognitive functioning, impulsive behavior, and substance use

**Figure 2:**
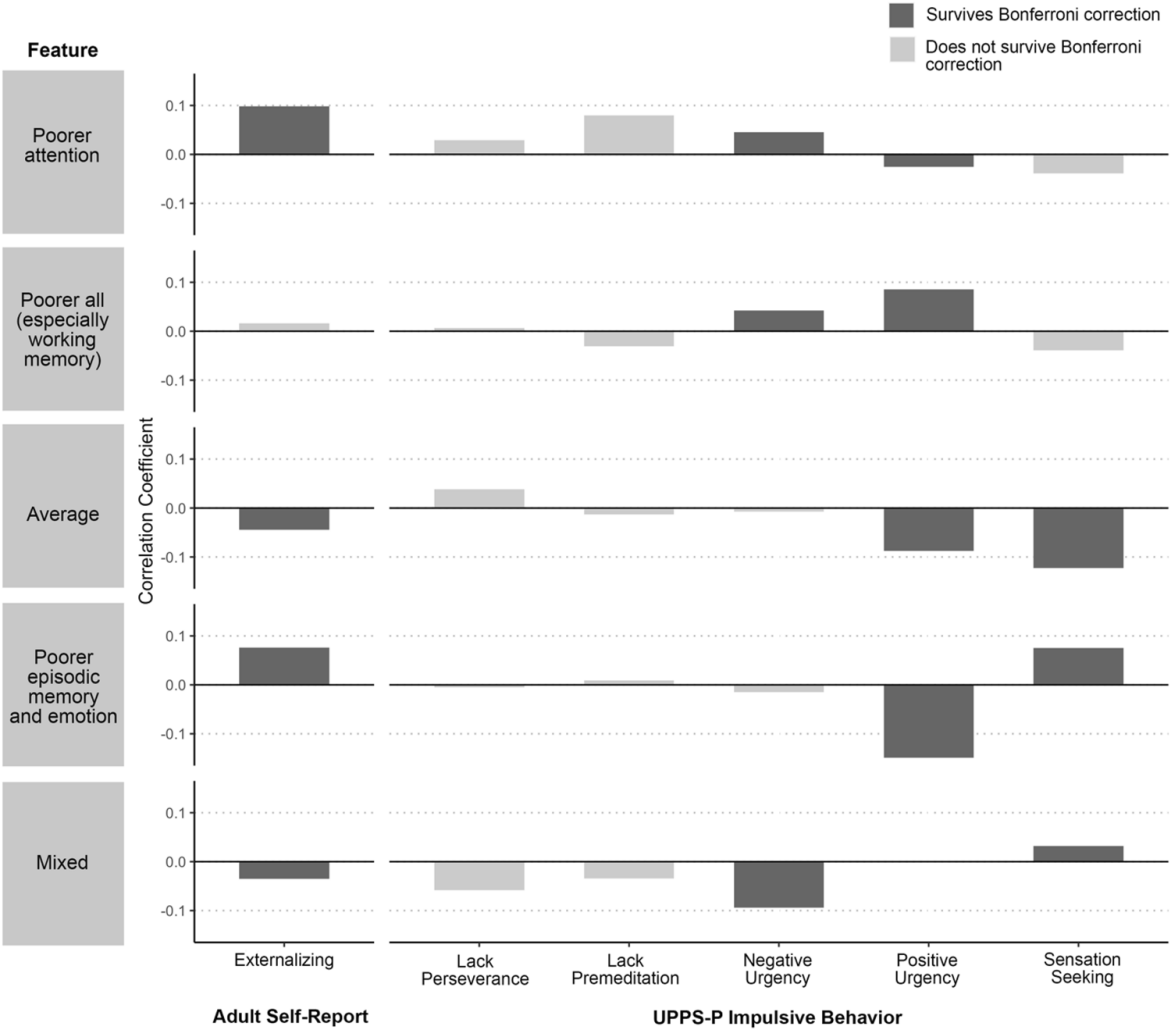
Results of dependent correlations with continuous feature values and risky/impulsive behavior outcomes. Bar plots showing correlation coefficient magnitudes for the relationship between continuous values for the five most populous features and risky/impulsive behavior outcomes.

**Figure 3:**
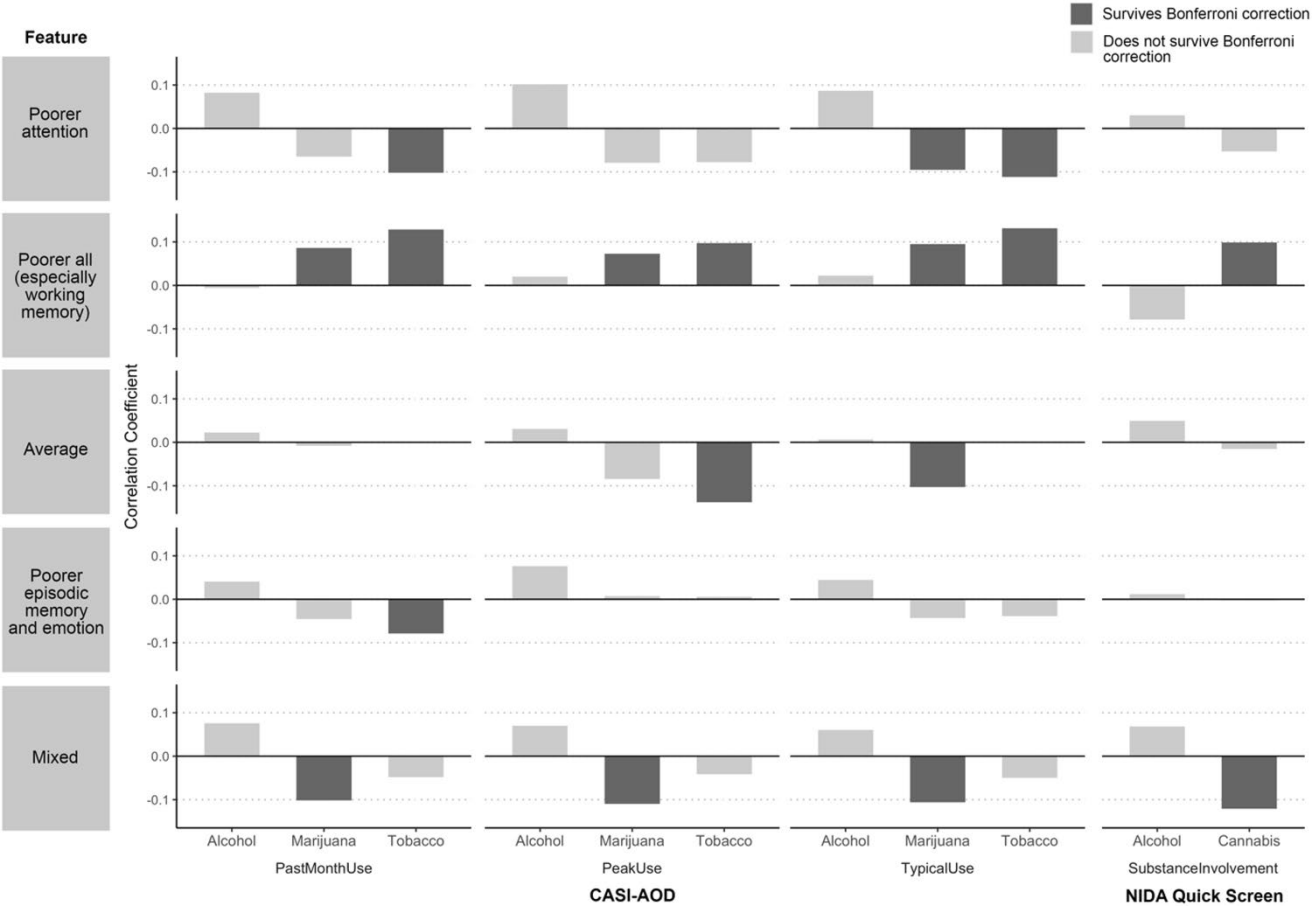
Results of dependent correlations with continuous feature values and substance use outcomes. Bar plots showing correlation coefficient magnitudes for the relationship between continuous values for the five most populous features and substance use outcomes. *CASI-AOD* Comprehensive Addiction Severity Index—Alcohol and Other Drugs, *NIDA* National Institute on Drug Abuse.

Relationships among feature values and outcomes are detailed in Table 2 and summarized in Figs. 2 and 3 (see Fig. S1b for zero-order correlations among single neurocognitive variables and outcomes).

**Table 2:** Results of dependent correlations with continuous feature values, risky/impulsive behavior, and substance use outcomes.

Outcome measure	Poorer attention 1	Poorer all (especially working memory) 2	Average 3	Poorer episodic memory and emotion 4	Mixed 5	Correlation comparison	*p*
Impulsive behavior outcomes							
Adult Self-Report							
Externalizing	0.10	0.02	− 0.04	0.08	− 0.04	1 vs. 3	0.007
						1 vs. 5	0.02
						4 vs. 3	0.02
UPPS-P							
Lack of Perseverance	0.03	0.01	0.04	− 0.005	− 0.06	–	–
Lack of Premeditation	0.08	− 0.03	− 0.01	0.01	− 0.03	–	–
Negative Urgency	0.05	0.04	− 0.01	− 0.01	− 0.09	1 vs. 5	0.01
						2 vs. 5	0.01
Positive Urgency	− 0.03	0.09	− 0.09	− 0.15	− 0.15	1 vs. 5	0.02
						2 vs. 3	0.002
						2 vs. 4	< 0.001
						2 vs. 5	< 0.001
Sensation Seeking	− 0.04	− 0.04	− 0.12	0.08	0.03	4 vs. 3	< 0.001
						5 vs. 3	0.01
Substance use outcomes							
CASI-AOD							
Past Month Use							
Alcohol	0.08	− 0.01	0.02	0.04	0.08	–	–
Cannabis	− 0.06	0.09	− 0.01	− 0.05	− 0.10	2 vs. 5	0.001
Tobacco	− 0.10	0.13	− 0.15	− 0.08	− 0.05	2 vs. 1	0.002
						2 vs. 3	< 0.001
						2 vs. 4	0.005
Peak Use							
Alcohol	0.10	0.02	0.03	0.08	0.07	–	–
Cannabis	− 0.08	0.07	− 0.08	0.01	− 0.11	2 vs. 5	0.012
Tobacco	− 0.08	0.10	− 0.14	0.01	− 0.04	2 vs. 3	0.002
Typical Use							
Alcohol	0.09	0.02	0.01	0.04	0.06	–	–
Cannabis	− 0.10	0.09	− 0.10	− 0.04	− 0.11	2 vs. 1	0.008
						2 vs. 3	0.006
						2 vs. 5	0.005
Tobacco	− 0.11	0.13	− 0.17	− 0.04	− 0.05	2 vs. 1	0.001
						2 vs. 3	< 0.001
NIDA Quick Screen							
Alcohol	0.03	− 0.08	0.05	0.01	0.07	–	–
Cannabis	− 0.05	0.10	− 0.02	− 0.002	− 0.12	2 vs. 5	< 0.001

*CASI-AOD* Comprehensive Addiction Severity Index—Alcohol and Other Drugs, *NIDA* National Institute on Drug Abuse.

#### Dependent correlations with risky/impulsive behavior outcomes

The feature characterized by poorer neurocognitive functioning (particularly working memory) correlated with increased impulsivity in affective circumstances (UPPS-Negative Urgency, UPPS-Positive Urgency). Specifically, the poorer overall neurocognitive functioning feature positively correlated with UPPS-Negative Urgency relative to the feature characterized by mixed neurocognitive functioning, as well as UPPS-Positive Urgency relative to the average feature, the feature characterized by poorer episodic memory and emotion identification, and the feature characterized by mixed neurocognitive functioning.

Additionally, the feature characterized by poorer episodic memory and emotion identification correlated with increased ASR-externalizing and UPPS-Sensation Seeking relative to the average feature. The feature characterized by poorer attention correlated with increased ASR-externalizing relative to the average feature and feature characterized by mixed functioning. Additionally, this feature was associated with increased UPPS-Negative Urgency but decreased UPPS-Positive Urgency relative to the feature characterized by mixed functioning.

Lastly, the feature characterized by mixed neurocognitive functioning correlated with increased UPPS-Sensation Seeking relative to the average feature. See Fig. S4 and Table S5 for analyses with internalizing *t-*score.

#### Dependent correlations with substance use outcomes

The feature characterized by poorer neurocognitive functioning (particularly working memory) correlated with increased CASI-AOD past-month, CASI-AOD peak, and CASI-AOD typical cannabis use relative to the feature characterized by mixed neurocognitive functioning. Additionally, the poorer neurocognitive functioning feature was associated with increased CASI-AOD past-month, peak and CASI-AOD typical tobacco use relative to the average feature. Finally, the poorer neurocognitive functioning feature correlated with increased NIDA Quick Screen problem use of cannabis relative to the feature characterized by mixed neurocognitive functioning.

The other four features (poorer attention; average; poorer episodic memory and emotion identification; mixed) generally demonstrated negative associations across cannabis and tobacco use outcomes.

## Discussion

Substantial research links neurocognitive deficits to risky and impulsive behaviors. However, previous work has been limited by the use of data and analytic tools that assume subfunctions of neurocognition are separate constructs, ultimately limiting our understanding of neurocognition as it operates within individuals and informs their behavior. The present study extends our understanding of neurocognitive function as it relates to risky and impulsive behaviors via a novel application of a Bayesian non-parametric latent feature learning method—IBP—to multiple neurocognitive tasks in a neurotypical sample. We showed that IBP estimates individual variability across and within subfunctions of neurocognition and that the resulting features meaningfully related to risky and impulsive behaviors. These within-individual features more precisely captured the association between cognition and behavior than the use of single measures from different neurocognitive tasks or the use of latent profile analysis (see Supplemental Materials for comparison analyses). Together, the present results suggest that variations in neurocognition, even within a relatively normative range, can be estimated. Further, these subtle variations meaningfully relate to real-world behaviors, even if those behaviors are not extreme or reflective of pathological behavior.

Different patterns of neurocognitive function were represented across the five IBP-derived features. Unsurprisingly, based on the present sample being drawn from a more neurotypical population, the most common feature was one that captures relatively average neurocognitive functioning across tasks. Four other features varied subtly in their relative neurocognitive function. Although the range of these relative variations were considered normative (i.e., within one standard deviation of the mean), our findings show that the IBP offers a sensitive approach to capturing nuances in neurocognitive functioning. In the present study, individuals best characterized by the average feature may have engaged all subfunctions of neurocognition when encountering information, supporting appropriate planning, organizing, and updating of behavior across experimental contexts. Other individuals, such as those represented by the feature characterized by relatively poorer functioning (particularly working memory), may have relied more heavily on non-verbal cues when attending to information, identifying errors, or representing and retrieving information from memory. Such reliance may contribute to variation in performance across language-based tasks (e.g., poorer performance on the D-KEFS Color-Word Interference, but no relative difficulty on D-KEFS Design Fluency). Thus, the application of IBP to a variety of neurocognitive tasks furthers our understanding of how neurocognitive function operates within individuals by richly capturing variability in how individuals engage with content (e.g., types of information) across contexts (e.g., different tasks).

The IBP-derived features showed associations with general and specific forms of risky and impulsive behaviors. The direct estimation of neurocognitive functioning across tasks and the relation to different subtypes of risky and impulsive behaviors within the same person and sample is a notable advance of the present approach over previous work. The feature characterized by poorer neurocognitive functioning in general (especially working memory) correlated with increased impulsivity in affective circumstances (i.e., positive and negative urgency), substance use patterns (i.e., past month, peak, and typical use), and substance use problems. These results are consistent with previous research noting relationships among poorer working memory, error detection, and inhibition and substance use problems^65^, substance use disorders^66^, as well as impulsive behavior in affective circumstances^23^. Such findings indicate that information critical for regulated behaviors may not be adequately processed or represented for some individuals, contributing to risky and impulsive behavior. In contrast, the feature characterized by poorer episodic memory and emotion correlated with greater trait externalizing and sensation-seeking and decreased positive urgency. Difficulty with contextualized memory and in interpreting social cues may lead to misperceptions of risk or consequence, contributing to increased impulsive behaviors broadly^67–69^. The feature characterized by poorer attention correlated with increased trait externalizing and negative urgency, as well as decreased positive urgency and substance use (see also Morris 2014)^70,71^. This relationship may suggest that relative difficulty shifting attention may leave individuals more susceptible to the salience of negative emotions and lead to generally dysregulated behavior^72^. Finally, the features characterized by average or mixed neurocognition demonstrated largely negative correlations with risky and impulsive outcomes, suggesting that neurocognitive patterns that displayed more average or distributed differences were not as relevant for risky and impulsive outcomes.

Together, variability in how individuals process and represent information appears to be important for specific expressions of risky and impulsive behaviors. Further, the difficulties in neurocognition need not be extreme to meaningfully relate to risky and impulsive behaviors. The specificity with which IBP can parse neurocognitive function is important because it allows us to clarify how relative variability across subfunctions may inform different expressions of risky and impulsive behaviors.

Several limitations of the present study should be noted. First, though a strength of IBP is its ability to retain individual variability in neurocognitive patterns through combinations of continuously valued features, it is possible that these combinations are too individual-specific to yield generalizable conclusions. Several features (Table S2) were sampled by one or two individuals and thus could not be used in aggregate analyses. It is possible that this individual variability really could be noise. Other analytic approaches may address noise by collapsing factors, exaggerating differences between latent profiles, or adopting hierarchical modeling frameworks. With IBP, noise may be captured in infrequently sampled features. To address whether such infrequently sampled features constitute noise or meaningful information, future research can utilize all features in a predictive framework (e.g., machine learning; longitudinal data)^73,74^. Second, while a strength of IBP is its flexibility—that is, the method is designed to adaptively characterize the data in hand—further work is needed to explore the stability of these neurocognitive features (see Supplemental Material for evidence of stability in the present sample) and the generalizability of the identified latent features for other data types and samples. Finally, and relatedly, the sample used for the present study was considered a “healthy” sample. The restricted range of scores might have limited the ability to identify potentially more clinically meaningful effects. It will be important to test the boundaries of applying IBP to neurocognitive data by examining its validity in clinical samples or samples that show more extreme atypicalities in neurocognitive functioning. However, our findings highlight the potential of this novel approach for studying neurocognitive dysfunction in populations that engage in more severe forms of impulsive and risky behavior.

The present study showed that IBP captured nuanced patterns of functioning across subfunctions of neurocognition and that these patterns differentially correlated with risky and impulsive behaviors. In the real world, it is not just people with substantial deficits in neurocognition that engage in risky and impulsive behaviors. Estimating neurotypical variation in neurocognition is crucial to providing a better understanding of its influence across a continuum of risky and impulsive behaviors.

## Data Availability

Data from the Nathan Kline Institute Rockland Sample may be accessed after completing a Data Use Agreement (DUA). Full steps for completing the DUA, accessing and downloading data are detailed here; https://fcon_1000.projects.nitrc.org/indi/enhanced/phenotypicdata.html.

## Supplementary Informatio﻿n

Supplementary Information.
