# Supplementary material for: Distinct neurocognitive fingerprints reflect differential associations with risky and impulsive behavior in a neurotypical sample
Source: Sci Rep. 2023 Jul 21;13:11782. doi: 10.1038/s41598-023-38991-0 (PMC10362008; doi:10.1038/s41598-023-38991-0)
Supplement: Supplementary file 1 — Supplementary Information. [file 41598_2023_38991_MOESM1_ESM.docx]

**Distinct Neurocognitive Fingerprints Reflect Differential Associations with Risky and Impulsive Behavior in a Neurotypical Sample**

**Supplemental Material**

**Comparing the Indian Buffet Process to Latent Profile Analysis**

To determine the utility of the Indian Buffet Process (IBP) in capturing individual variability in neurocognitive function, we performed a comparison analysis using a traditional dimension reduction method, latent profile analysis (LPA). Briefly, LPA estimates distinct profiles that represent variability across measures of interest (e.g., measures of neurocognitive function), and assigns individuals to the profile for which their likelihood of belonging (i.e., their conditional probability) is largest.

We applied LPA to the same 27 z-scored neurocognitive measures for the full sample (*n* = 673) using the R package *tidylpa*, which uses the expectation-maximization algorithm via the package *mclust* to determine the maximum likelihood estimates for model parameters ^1-3^. We included a single imputation for missing data and evaluated one- through eleven- class (i.e., profile) models. To select the optimal number of profiles, comparative model fit was assessed using the Bayesian Information Criterion, where smaller values indicate better fit ^4^; entropy, where larger values (values range from 0-1; values > .8 are considered acceptable) indicate greater classification certainty and class discrimination ^5^; bootstrapped likelihood ratio tests, where a significant *p*-value indicates that a model with *k* profiles has preferable fit relative to a model with *k* – 1 profiles; and profile size, where profiles with counts greater than 5% of the total sample size (*n* = 33) were considered representative of the profile ^6^. Table S6 summarizes model fits, with the optimal model bolded.

Results indicated that the six-class solution was the best fitting model because it demonstrated a lower BIC value (49,209.11) than the one- through five-class and seven- through eleven-class solutions, as well as greater classification certainty (entropy = 0.89) than the seven- through eleven- class solutions. The first profile was characterized by generally average function across subfunctions and better error detection; the second profile was characterized by better function across subfunctions; the third profile was characterized by the poorest function across subfunctions, with several measures exceeding one standard deviation lower than the mean; the fourth profile was characterized by poor function across subfunctions and average attention; the fifth profile was characterized by average function and slightly poor cognitive flexibility; and the sixth profile was characterized by average function and very poor error detection (two standard deviations below the mean; Figure S5).

We contrasted the profile assignments from LPA with the IBP-derived features (Figure S6). Though many individuals are classified as performing very poorly or poorly by their assigned profile (Profile 3, *n* = 51; and Profile 4, *n* = 44), most of the profiles are captured within the “Average” feature estimated by IBP (Profile 1, *n* = 106, Profile 2, *n* = 237, Profile 3, *n* = 44, Profile 4, *n* = 38, Profile 5, *n* = 98, Profile 6, *n* = 76). We interpret this discrepancy as arising from the priorities and assumptions of LPA. Because LPA constrains variance within profiles to reduce the number of model parameters (and sample size), it often produces homogenous classes that differ maximally but perhaps whose differences are inflated ^7^.

**Modeling Solution Sensitivity Analysis**

While a strength of IBP is its flexibility in adapting to the data, the stability of IBP-derived feature solutions is one limitation^8^. To determine whether our final modeling solution was robust, we conducted sensitivity analyses using a split-half approach. Half the participants (N = 337) from the full sample were randomly selected using the sample_frac command in R^9^. IBP was applied to the z-scores from their 27 neurocognitive measures with the concentration parameter *α* initialized at five. For each participant, we extracted the continuous feature values corresponding to their sampled latent features (*K*) in the final of 10 iterations. *K* stabilized at 25; the concentration parameter *α* remained between 1.9 and 2.3 and stabilized at 1.9; the variance of observed data parameter *σ_X_* stabilized at 0.8; and the variance of posterior mean weights parameter *σ_A_* stabilized at 0.5 (Figure S7). We selected features with at least 17 people (i.e., 5% of the sample) to examine in analyses. Four features met this criterion: (i) One feature was characterized by relatively poorer working memory; (ii) one feature was characterized by relatively poorer attention; (iii) one feature captured mixed functioning, with slightly poorer emotion identification and sensorimotor speed; and (iv) one feature reflected average functioning across neurocognitive subfunctions (Figure S8). Though the number of features differed (four in the half-sample, and five in the full sample), the neurocognitive patterns represented by IBP-derived features in the full sample were conceptually represented when using a subset of the sample (particularly poorer working memory, poorer attention performance, poorer emotion identification, and average functioning across subfunctions). We attribute differences in the number of features to decreases in sample size^10^.

**Supplemental References**

1 R Core Team. R: A langugage and environment for statistical computing. (2022).

2 Rosenberg, J. M., Beymer, P. N., Anderson, D. J., Van Lissa, C. J. & Schmidt, J. A. tidyLPA: An R Package to Easily Carry Out Latent Profile Analysis (LPA) Using Open-Source or Commercial Software. *Journal of Open Source Software* **3**, 978 (2018). <https://doi.org:https://doi.org/10.21105/joss.00978>

3 Scrucca, L., Fop, M., Murphy, T. B. & Raftery, A. E. mclust 5: clustering, classification and density estimation using Gaussian finite mixture models. *The R Journal* **8**, 289-317 (2016). <https://doi.org:https://doi.org/10.32614/RJ-2016-021>

4 Schwarz, G. Estimating the dimension of a model. *The Annals of Statistics* **6**, 461–464 (1978). <https://doi.org:https://doi.org/10.1214/aos/1176344136>

5 Celeux, G. & Soromenho, G. An entropy criterion for assessing the number of clusters in a mixture model. *Journal of Classification* **13**, 195-212 (1996). <https://doi.org:https://doi.org/10.1007/BF01246098>

6 Nylund, K. L., Asparouhov, T. & Muthén, B. O. Deciding on the number of classes in latent class analysis and growth mixture modeling: A Monte Carlo simulation study. *Structural Equation Modeling* **14**, 535-569 (2007). <https://doi.org:https://doi.org/10.1080/10705510701575396>

7 Lubke, G. & Neale, M. C. Distinguishing between latent classes and continuous factors: Resolution by maximum likelihood? *Multivar Behav Res* **41**, 499-532 (2006). <https://doi.org:https://doi.org/10.1207/s15327906mbr4104_4>

8 Doshi-Velez, F. The Indian buffet process: Scalable inference and extensions. *Master's thesis, University of Cambridge* (2009).

9 Wickham, H., François, R., Henry, L., Müller, K. & Vaughan, D. *dplyr: A Grammar of Data Manipulation*, <<https://CRAN.R-project.org/package=dplyr>> (2023).

10 Griffiths, T. L. & Ghahramani, Z. The Indian Buffet Process: An Introduction and Review. *J Mach Learn Res* **12**, 1185-1224 (2011). <https://doi.org:https://doi.org/10.5555/1953048.2021039>

11 Wei, T. & Simko, V. *R package 'corrplot': Visualization of a Correlation Matrix*, <<https://github.com/taiyun/corrplot>> (2021).

**Supplemental Figures**

**Figure S1**. Heatmap showing Spearman correlations across (a) Demographic variables, neurocognitive measures, and continuous feature values, and (b) Neurocognitive measures and impulsive behavior and substance use outcomes.


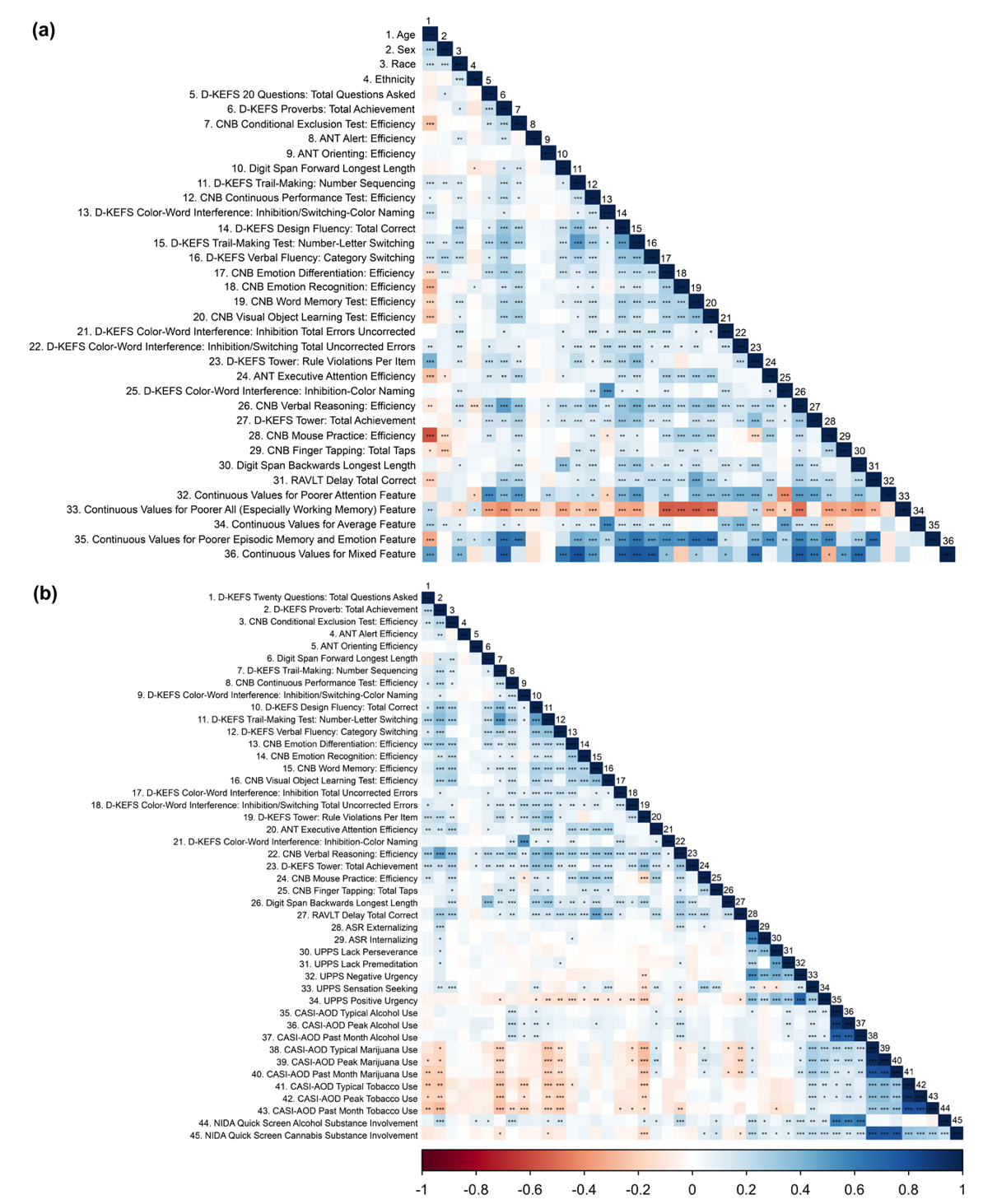


*Note.* Heatmap was made using the R package *corrplot* ^11^. ANT = Attention Network Test; CASI-AOD = Comprehensive Addiction Severity Index – Alcohol and Other Drugs; CNB = Penn Computerized Neurocognitive Battery; D-KEFS = Delis-Kaplan Executive Function System; NIDA = National Institute on Drug Abuse. * p < .05, ** p < .01, *** p < .001.

**Figure S2.** Density plots showing distributions for the impulsive behavior and substance use outcomes.


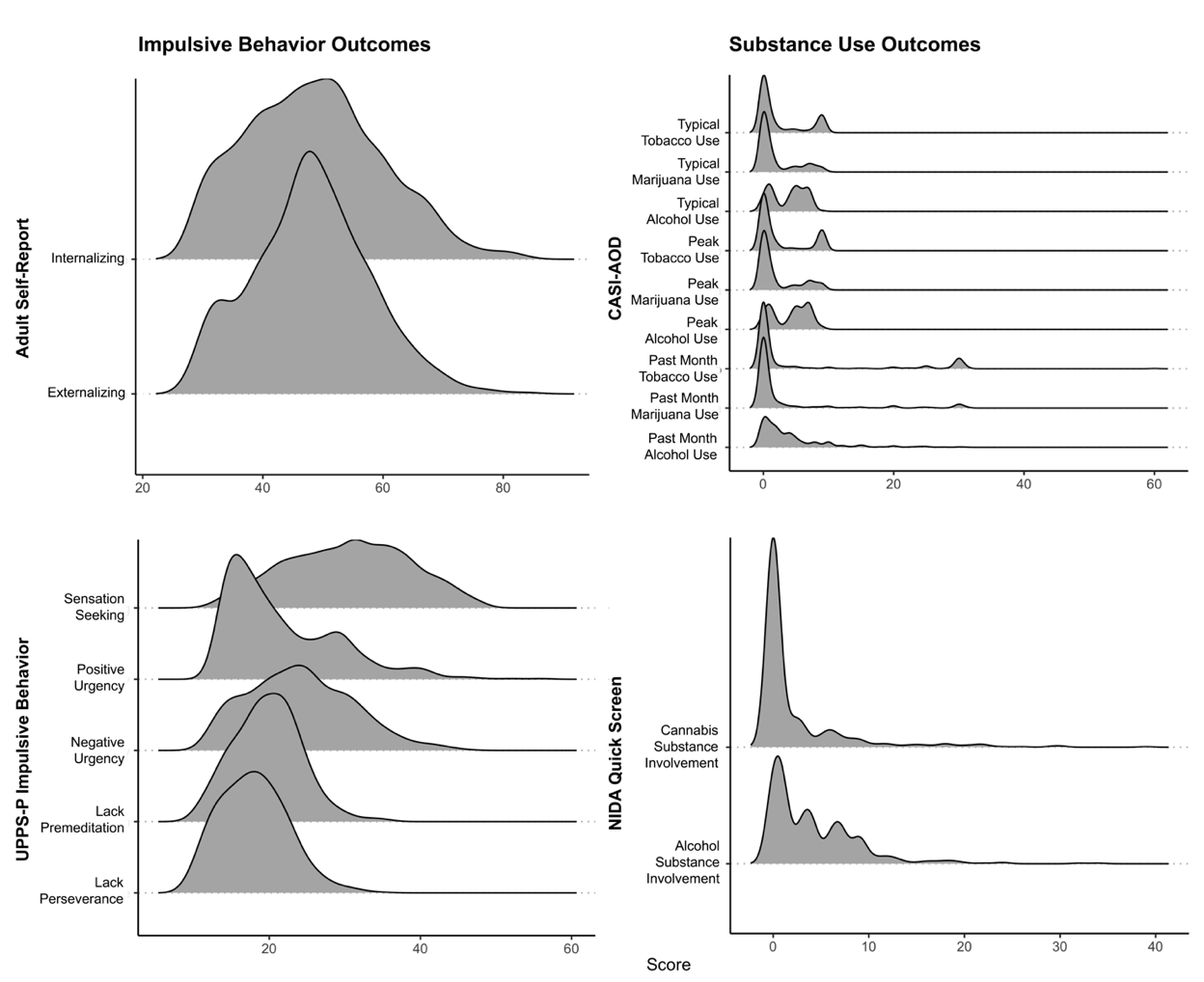


*Note.* CASI-AOD = Comprehensive Addiction Severity Index – Alcohol and Other Drugs; NIDA = National Institute on Drug Abuse.

**Figure S3.** Traceplots for IBP parameters *K*, *α*, *σ_X_*, and *σ_A_*.


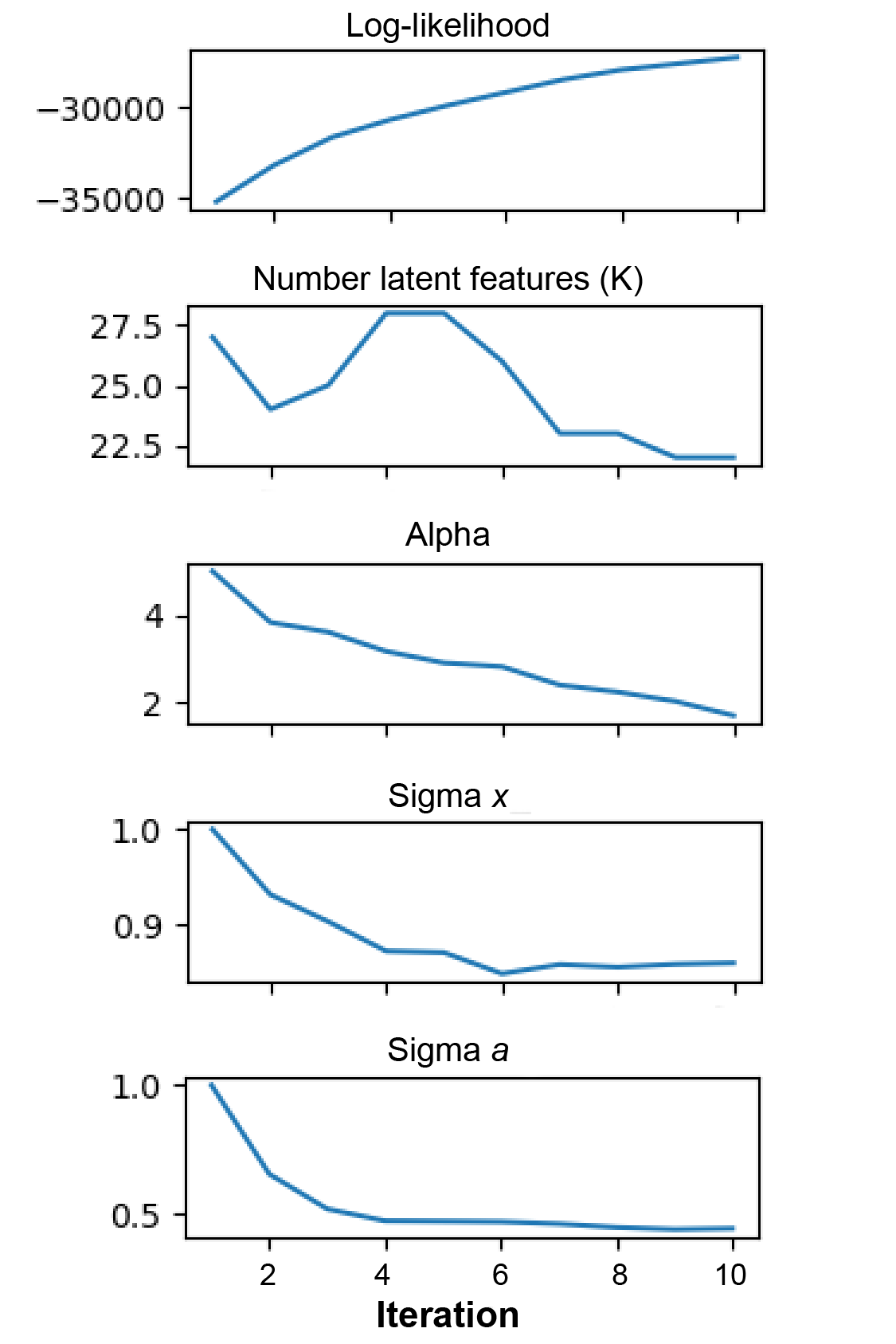


*Note.* The traceplots demonstrate model convergence: *K*, the number of latent features, oscillates between 22 and 23; the concentration parameter *α* remains between 1.7 and 2.3; *σ_X_* (the variance of the observed data) stabilizes at 0.9; *σ_A_* (the variance of the posterior mean weights) stabilizes at 0.4. We selected the sampled *K* number of latent features and extracted the corresponding continuous feature values present in the final iteration (*n* = 10).

**Figure S4.** Results of Dependent Correlations with Continuous Feature Values and Internalizing


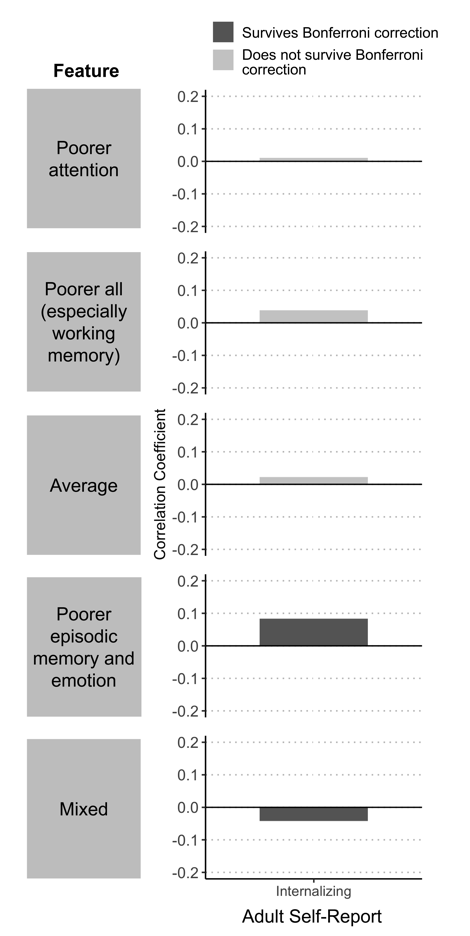


*Note.* Bar plots showing correlation coefficient magnitudes for the relationship between continuous values for the five most populous features and internalizing *t*-scores.

**Figure S5.** Neurocognitive patterns captured within each latent profile.


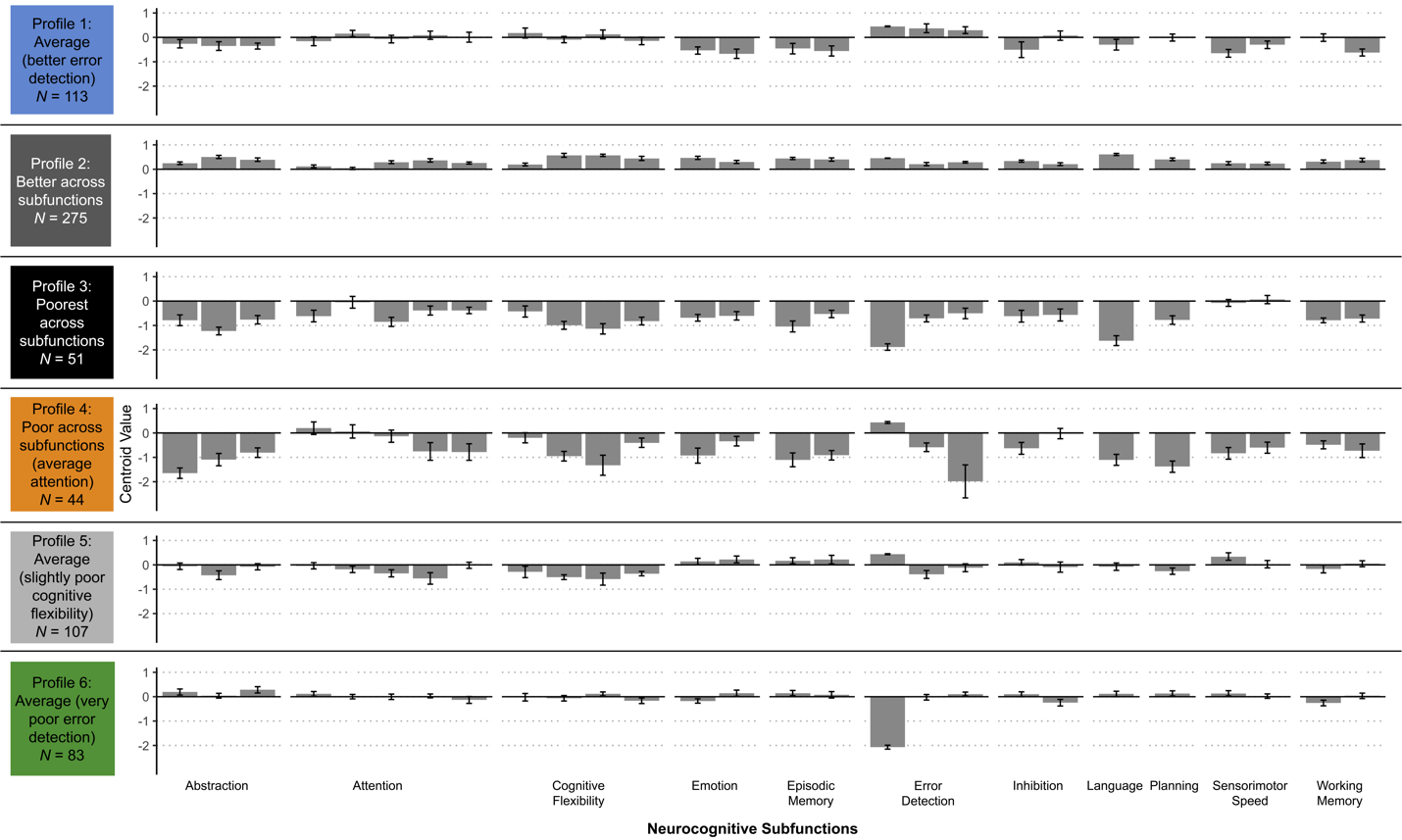


*Note*. Bar plot showing profile centroids and standard errors within each of the six profiles for the 27 neurocognitive measures and counts for individuals most likely to be classified into each profile.

**Figure S6.** Classification comparison between latent profile analysis assigned profiles and Indian Buffet Process sampled features.


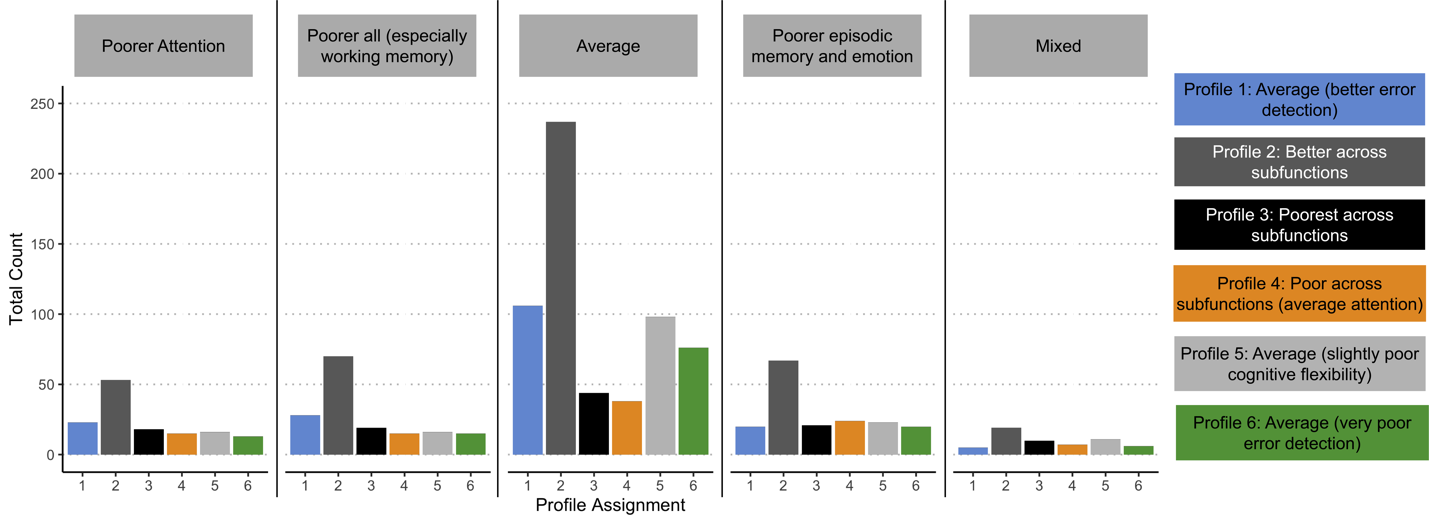


*Note*. Bar plot showing counts for assigned profiles and latent features derived from latent profile analysis and the Indian Buffet Process, respectively. Though many individuals are classified as performing very poorly or poorly by their assigned profile (Profiles 3 and 4), most of the sample is captured within the “Average” feature estimated by the Indian Buffet Process.

**Figure S7.** Traceplots for IBP parameters *K*, *α*, *σ_X_*, and *σ_A_* from half-sample analysis.

**
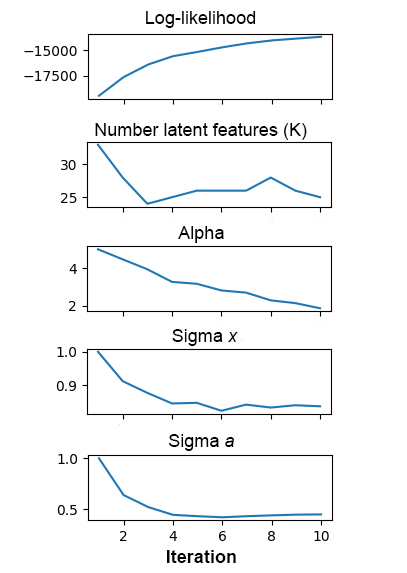
**

*Note.* The traceplots demonstrate model convergence: *K*, the number of latent features, oscillates between 24 and 26; the concentration parameter *α* remains between 1.9 and 2.3; *σ_X_* (the variance of the observed data) stabilizes at 0.8; *σ_A_* (the variance of the posterior mean weights) stabilizes at 0.5. We selected the sampled *K* number of latent features and extracted the corresponding continuous feature values present in the final iteration (*n* = 10).

**Figure S8.** Neurocognitive patterns represented by latent features in half the sample.


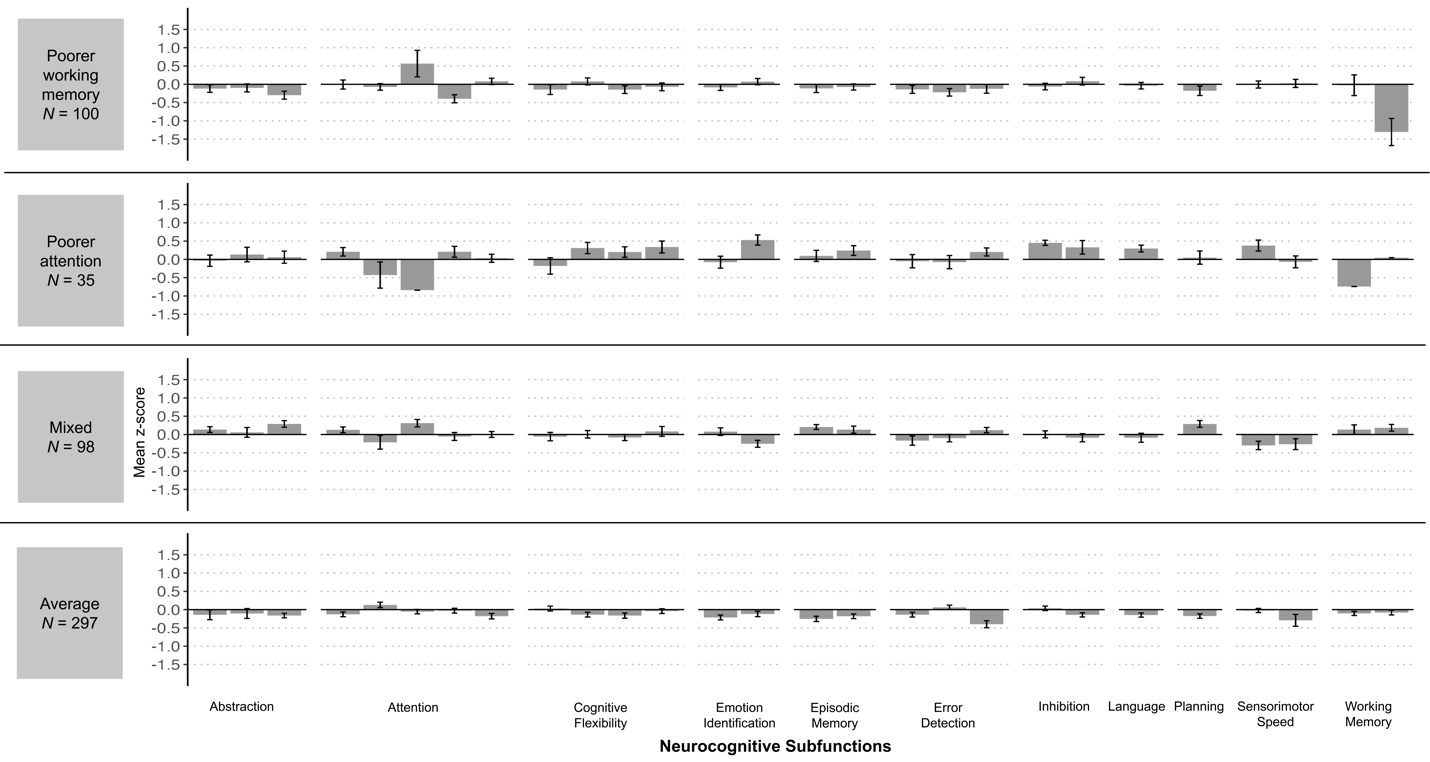


*Note*. Mean z-score and counts for each of the 27 neurocognitive measures across individuals who sampled each latent feature. Individual measures corresponding to each subfunction, ordered left to right, are as follows: Abstraction (D-KEFS Twenty Questions: Total Questions Asked; D-KEFS Proverb: Total Achievement; CNB Conditional Exclusion Test: Efficiency), attention (ANT Alert Efficiency; ANT Orienting Efficiency; Digit Span Forward Longest Length; D-KEFS Trail-Making: Number Sequencing; CNB Continuous Performance Test: Efficiency), cognitive flexibility (D-KEFS Color-Word Interference: Inhibition/Switching-Color Naming; D-KEFS Design Fluency: Total Correct; D-KEFS Trail-Making Test: Number-Letter Switching; D-KEFS Verbal Fluency: Category Switching), emotion (CNB Emotion Differentiation: Efficiency; CNB Emotion Recognition: Efficiency), episodic memory (CNB Word Memory: Efficiency; CNB Visual Object Learning Test: Efficiency), error detection (D-KEFS Color-Word Interference: Inhibition Total Errors Uncorrected; D-KEFS Color-Word Interference: Inhibition/Switching Total Uncorrected Errors; D-KEFS Tower: Rule Violations Per Item), inhibition (ANT Executive Attention Efficiency; D-KEFS Color-Word Interference: Inhibition-Color Naming), language (CNB Verbal Reasoning: Efficiency), planning (D-KEFS Tower: Total Achievement), sensorimotor speed (CNB Mouse Practice: Efficiency; CNB Finger Tapping: Total Taps), working memory (Digit Span Backwards Longest Length; RAVLT Delay Total Correct). D-KEFS = Delis-Kaplan Executive Function System; ANT = Attention Network Test; CNB = Penn Computerized Neurocognitive Battery; RAVLT = Rey Auditory Verbal Learning Test.

**Supplemental Tables**

**Table S1**. Neurocognitive functioning, Impulsive behavior, and Substance Use outcomes measure completeness.

| Subfunction | Test | Count | % |
| --- | --- | --- | --- |
| Abstraction | D-KEFS Twenty Questions: Total Questions Asked | 375 | 56 |
|  | D-KEFS Proverb: Total Achievement | 369 | 55 |
|  | CNB Conditional Exclusion Test: Efficiency | 647 | 96 |
| Attention | ANT Alert Efficiency | 641 | 95 |
|  | ANT Orienting Efficiency | 642 | 95 |
|  | Digit Span Forward Longest Length | 464 | 69 |
|  | D-KEFS Trail-Making: Number Sequencing | 641 | 95 |
|  | CNB Continuous Performance Test: Efficiency | 647 | 96 |
| Cognitive Flexibility | D-KEFS Color-Word Interference: Inhibition/Switching-Color Naming | 631 | 93 |
|  | D-KEFS Design Fluency: Total Correct | 639 | 95 |
|  | D-KEFS Trail-Making Test: Number-Letter Switching | 641 | 95 |
|  | D-KEFS Verbal Fluency: Category Switching | 621 | 92 |
| Emotion | CNB Emotion Differentiation: Efficiency | 648 | 96 |
|  | CNB Emotion Recognition: Efficiency | 648 | 96 |
| Episodic Memory | CNB Word Memory: Efficiency | 648 | 96 |
|  | CNB Visual Object Learning Test: Efficiency | 648 | 96 |
| Error Detection | D-KEFS Color-Word Interference: Inhibition Total Errors Uncorrected | 633 | 94 |
|  | D-KEFS Color-Word Interference: Inhibition/Switching Total Uncorrected Errors | 631 | 94 |
|  | D-KEFS Tower: Rule Violations Per Item | 640 | 95 |
| Inhibition | ANT Executive Attention Efficiency | 640 | 95 |
|  | D-KEFS Color-Word Interference: Inhibition-Color Naming | 633 | 94 |
| Language | CNB Verbal Reasoning: Efficiency | 648 | 96 |
| Planning | D-KEFS Tower: Total Achievement | 640 | 95 |
| Sensorimotor Speed | CNB Mouse Practice: Efficiency | 648 | 96 |
|  | CNB Finger Tapping: Total Taps | 392 | 58 |
| Working Memory | Digit Span Backwards Longest Length | 464 | 69 |
|  | RAVLT Delay Total Correct | 447 | 66 |
| ASR | Externalizing *t*-score | 653 | 97 |
| UPPS-P Impulsive Behavior | Lack Perseverance | 656 | 97 |
|  | Lack Premeditation | 656 | 97 |
|  | Negative Urgency | 656 | 97 |
|  | Positive Urgency | 656 | 97 |
|  | Sensation Seeking | 656 | 97 |
| CASI-AOD | Past Month Use |  |  |
|  | Alcohol | 557 | 83 |
|  | Cannabis | 369 | 55 |
|  | Tobacco | 351 | 52 |
|  | Peak Use |  |  |
|  | Alcohol | 558 | 83 |
|  | Cannabis | 370 | 55 |
|  | Tobacco | 351 | 52 |
|  | Typical Use |  |  |
|  | Alcohol | 564 | 84 |
|  | Cannabis | 388 | 58 |
|  | Tobacco | 362 | 54 |
| NIDA Quick Screen | Alcohol Substance Involvement | 514 | 76 |
|  | Cannabis Substance Involvement | 528 | 78 |

*Note.* ANT = Attention Network Test; ASR = Adult Self-Report; CASI-AOD = Comprehensive Addiction Severity Index – Alcohol and Other Drugs; CNB = Penn Computerized Neurocognitive Battery; D-KEFS = Delis-Kaplan Executive Function System; NIDA = National Institute on Drug Abuse.

**Table S2.** Feature sampling counts.

| **Feature** | **Sampling count** | **Description** |
| --- | --- | --- |
| **1** | **138** | Poorer attention |
| 2 | 21 |  |
| **3** | **163** | Poorer all (especially working memory) |
| **4** | **599** | Average |
| 5 | 2 |  |
| **6** | **175** | Poorer episodic memory and emotion |
| 7 | 3 |  |
| **8** | **58** | Mixed |
| 9 | 2 |  |
| 10 | 1 |  |
| 11 | 3 |  |
| 12 | 1 |  |
| 13 | 2 |  |
| 14 | 3 |  |
| 15 | 2 |  |
| 16 | 2 |  |
| 17 | 1 |  |
| 18 | 1 |  |
| 19 | 5 |  |
| 20 | 1 |  |
| 21 | 3 |  |
| 22 | 3 |  |

*Note.* Features included for group analysis are bolded. For the neurocognitive pattern represented by the second feature (*n* = 21), see Figure S4.

**Table S3.** Patterns of neurocognitive performance for each feature.

| Subfunction | **Feature** | **Poorer attention** | | **Poorer all (especially working memory)** | | **Average** | | **Poorer episodic memory and emotion** | | **Mixed** | |
| --- | --- | --- | --- | --- | --- | --- | --- | --- | --- | --- | --- |
|  | Test | Mean | *SE* | Mean | *SE* | Mean | *SE* | Mean | *SE* | Mean | *SE* |
| Abstraction | D-KEFS Twenty Questions: Total Questions Asked | -0.05 | 0.10 | -0.13 | 0.09 | 0.05 | 0.05 | Not sampled | – | Not sampled | – |
|  | D-KEFS Proverb: Total Achievement | 0.06 | 0.09 | -0.18 | 0.09 | -0.10 | 0.06 | Not sampled | – | Not sampled | – |
|  | CNB Conditional Exclusion Test: Efficiency | 0.17 | 0.08 | -0.25 | 0.09 | 0.01 | 0.04 | 0.03 | 0.08 | -0.54 | 0.15 |
| Attention | ANT Alert Efficiency | 0.02 | 0.10 | 0.08 | 0.08 | -0.08 | 0.05 | 0.05 | 0.07 | -0.18 | 0.27 |
|  | ANT Orienting Efficiency | 0.06 | 0.07 | -0.40 | 0.12 | 0.09 | 0.05 | 0.05 | 0.09 | 0.08 | 0.13 |
|  | Digit Span Forward Longest Length | -0.31 | 0.21 | 0.14 | 0.21 | -0.02 | 0.05 | -0.05 | 0.08 | -0.09 | 0.14 |
|  | D-KEFS Trail-Making: Number Sequencing | -0.39 | 0.09 | -0.11 | 0.09 | 0.03 | 0.04 | -0.08 | 0.09 | 0.06 | 0.17 |
|  | CNB Continuous Performance Test: Efficiency | -0.07 | 0.10 | 0.03 | 0.07 | -0.03 | 0.04 | -0.20 | 0.12 | -0.01 | 0.08 |
| Cognitive Flexibility | D-KEFS Color-Word Interference: Inhibition/Switching-Color Naming | 0.04 | 0.08 | -0.19 | 0.09 | 0.07 | 0.04 | 0.00 | 0.08 | 0.07 | 0.17 |
|  | D-KEFS Design Fluency: Total Correct | 0.07 | 0.08 | -0.01 | 0.08 | -0.05 | 0.04 | -0.18 | 0.09 | 0.14 | 0.21 |
|  | D-KEFS Trail-Making Test: Number-Letter Switching | -0.18 | 0.09 | -0.32 | 0.10 | 0.02 | 0.04 | -0.09 | 0.09 | -0.19 | 0.19 |
|  | D-KEFS Verbal Fluency: Category Switching | -0.09 | 0.08 | -0.12 | 0.07 | -0.06 | 0.04 | 0.12 | 0.08 | 0.51 | 0.16 |
| Emotion | CNB Emotion Differentiation: Efficiency | 0.08 | 0.08 | -0.07 | 0.08 | 0.00 | 0.05 | -0.06 | 0.08 | -0.06 | 0.14 |
|  | CNB Emotion Recognition: Efficiency | 0.17 | 0.08 | 0.11 | 0.08 | -0.05 | 0.05 | -0.39 | 0.08 | -0.13 | 0.12 |
| Episodic Memory | CNB Word Memory: Efficiency | -0.05 | 0.10 | 0.06 | 0.07 | -0.03 | 0.04 | -0.26 | 0.09 | -0.41 | 0.19 |
|  | CNB Visual Object Learning Test: Efficiency | 0.12 | 0.08 | -0.05 | 0.08 | 0.01 | 0.04 | -0.19 | 0.09 | -0.25 | 0.16 |
| Error Detection | D-KEFS Color-Word Interference: Inhibition Total Errors Uncorrected | 0.06 | 0.08 | -0.18 | 0.09 | -0.04 | 0.04 | 0.00 | 0.08 | 0.20 | 0.13 |
|  | D-KEFS Color-Word Interference: Inhibition/Switching Total Uncorrected Errors | 0.07 | 0.08 | -0.39 | 0.08 | -0.03 | 0.04 | 0.04 | 0.08 | 0.36 | 0.17 |
|  | D-KEFS Tower: Rule Violations Per Item | 0.00 | 0.10 | -0.04 | 0.06 | -0.09 | 0.05 | -0.18 | 0.11 | -0.01 | 0.14 |
| Inhibition | ANT Executive Attention Efficiency | 0.00 | 0.09 | -0.02 | 0.08 | -0.11 | 0.06 | -0.20 | 0.08 | -0.01 | 0.15 |
|  | D-KEFS Color-Word Interference: Inhibition-Color Naming | -0.09 | 0.09 | 0.12 | 0.08 | 0.06 | 0.04 | -0.06 | 0.08 | -0.29 | 0.20 |
| Language | CNB Verbal Reasoning: Efficiency | 0.02 | 0.09 | -0.08 | 0.08 | -0.07 | 0.05 | -0.07 | 0.08 | -0.30 | 0.16 |
| Planning | D-KEFS Tower: Total Achievement | -0.07 | 0.10 | -0.24 | 0.09 | 0.00 | 0.04 | -0.07 | 0.09 | -0.06 | 0.17 |
| Sensorimotor Speed | CNB Mouse Practice: Efficiency | 0.23 | 0.08 | -0.13 | 0.08 | 0.09 | 0.04 | -0.20 | 0.08 | -0.29 | 0.13 |
|  | CNB Finger Tapping: Total Taps | -0.06 | 0.11 | 0.05 | 0.08 | -0.05 | 0.06 | 0.17 | 0.17 | -0.23 | 0.21 |
| Working Memory | Digit Span Backwards Longest Length | 0.09 | 0.21 | -0.60 | 0.09 | -0.03 | 0.04 | 0.06 | 0.07 | -0.21 | 0.13 |
|  | RAVLT Delay Total Correct | 0.02 | 0.30 | -0.59 | 0.29 | 0.07 | 0.04 | -0.42 | 0.09 | 0.16 | 0.17 |

*Note.* D-KEFS = Delis-Kaplan Executive Function System; ANT = Attention Network Test; CNB = Penn Computerized Neurocognitive Battery; RAVLT = Rey Auditory Verbal Learning Test.

**Table S4.** Multi-feature sampling counts.

| **Feature** | **Sampling count** | **Description** |
| --- | --- | --- |
| 1, 4 | 53 | Poorer cognitive flexibility |
| 3, 4 | 73 | Poorer emotion |
| 4 | 233 | Average |
| 4, 6 | 80 | Poorer abstraction |
| 4, 8 | 21 | Better abstraction, attention, cognitive flexibility, planning, and working memory |
| 3, 4, 6 | 23 | Poorer abstraction, attention, and cognitive flexibility |
| 1, 4, 6 | 22 | Poorer attention, error detection, and language; better working memory |

*Note.* Counts for unique combinations of sampled features. Approximately 40% of participants sampled multiple features. Feature 4 (the “average” feature) is most often sampled with other features, which makes sense given that this is a neurotypical sample. This pattern of sampling suggests that in certain contexts or given certain experiences, some neurocognitive functions within individuals characterized by generally “average” neurocognition can relatively diverge from average.

**Table S5.** Results of Dependent Correlations with Internalizing.

| Adult Self-Report | Poorer Attention  1 | Poorer all (especially working memory)  2 | Average  3 | Poorer episodic memory and emotion  4 | Mixed  5 | Correlation Comparison | *p* |
| --- | --- | --- | --- | --- | --- | --- | --- |
| Internalizing | 0.01 | 0.04 | -0.04 | 0.08 | -0.04 | 4 vs. 5 | .02 |

**Table S6.** Model fit for the latent profile analysis.

| Classes | Log-likelihood | AIC | BIC | Entropy | BLRT | BLRT *p* |
| --- | --- | --- | --- | --- | --- | --- |
| 1 | -25,840.82 | 51,789.63 | 52,033.26 | 1.00 | – | – |
| 2 | -24,939.90 | 50,043.81 | 50,413.77 | 0.87 | 1,801.82 | .010 |
| 3 | -24,501.78 | 49,223.57 | 49,719.86 | 0.93 | 876.24 | .010 |
| 4 | -24,340.15 | 48,956.31 | 49,578.93 | 0.90 | 323.26 | .010 |
| 5 | -24,071.53 | 48,475.06 | 49,224.01 | 0.91 | 537.25 | .010 |
| **6** | **-23,972.91** | **48,333.83** | **49,209.11** | **0.89** | **197.23** | **.010** |
| 7 | -23,926.13 | 48,296.27 | 49,297.88 | 0.84 | 93.56 | .010 |
| 8 | -23,799.65 | 48,099.30 | 49,227.24 | 0.87 | 252.97 | .010 |
| 9 | -23,766.62 | 48,089.24 | 49,343.50 | 0.86 | 66.07 | .020 |
| 10 | -23,753.45 | 48,118.90 | 49,499.49 | 0.84 | 26.34 | .792 |
| 11 | -23,621.53 | 47,911.06 | 49,417.98 | 0.86 | 263.84 | .010 |

*Note*. The selected model (6-class) is bolded. AIC = Akaike Information Criterion; BIC = Bayesian Information Criterion; BLRT = bootstrapped likelihood ratio test.
